# Neuroprotective Studies of Evodiamine in an Okadaic Acid-Induced Neurotoxicity

**DOI:** 10.3390/ijms22105347

**Published:** 2021-05-19

**Authors:** Ching-Hsuan Chou, Chia-Ron Yang

**Affiliations:** School of Pharmacy, College of Medicine, National Taiwan University, Taipei 10050, Taiwan; d09423201@ntu.edu.tw

**Keywords:** Alzheimer’s disease, evodiamine, tau, okadaic acid, aggregation

## Abstract

Background: Alzheimer’s disease (AD) is the most common neurodegenerative disease, and it manifests as progressive memory loss and cognitive decline. However, there are no effective therapies for AD, which is an urgent problem to solve. Evodiamine, one of the main bioactive ingredients of *Evodia rutaecarpa*, has been reported to ameliorate blood–brain barrier (BBB) permeability and improve cognitive impairment in ischemia and AD mouse models. However, whether evodiamine alleviates tauopathy remains unclear. This study aimed to examine whether evodiamine ameliorates tau phosphorylation and cognitive deficits in AD models. Methods: A protein phosphatase 2A inhibitor, okadaic acid (OA), was used to induce tau phosphorylation to mimic AD-like models in neuronal cells. Protein expression and cell apoptosis were detected using Western blotting and flow cytometry, respectively. Spatial memory/cognition was assessed using water maze, passive avoidance tests, and magnetic resonance imaging assay in OA-induced mice models, and brain slices were evaluated further by immunohistochemistry. Results: The results showed that evodiamine significantly reduced the expression of phosphor-tau, and further decreased tau aggregation and neuronal cell death in response to OA treatment. This inhibition was found to be via the inhibition of glycogen synthase kinase 3β, cyclin-dependent kinase 5, and mitogen-activated protein kinase pathways. In vivo results indicated that evodiamine treatment ameliorated learning and memory impairments in mice, whereas Western blotting and immunohistochemical analysis of the mouse brain also confirmed the neuroprotective effects of evodiamine. Conclusions: Evodiamine can decrease the neurotoxicity of tau aggregation and exhibit a neuroprotective effect. Our results demonstrate that evodiamine has a therapeutic potential for AD treatment.

## 1. Introduction

Alzheimer’s disease (AD) is a common progressive neurodegenerative disease worldwide, representing a global health concern. Approximately 50 million individuals have dementia worldwide; this figure will almost triple by 2050 [1]. AD causes a progressive loss of mental capacity and behavior, causing a functional decline in the ability to learn [2]. The causes of AD are unknown; however, factors such as oxidative stress and neuro-inflammation contribute to the pathogenesis of AD [2]. Four drugs are approved by the FDA for cognitive manifestations of AD treatment: the acetylcholine esterase inhibitors rivastigmine, galantamine, and donepezil elevate the acetylcholine levels in the brain nerves and the NMDA receptor antagonist memantine protects the nervous tissues against glutamate-mediated cytotoxicity. These agents provide symptomatic relief; however, the benefits remain limited [3,4]. Therefore, the development of new drugs for AD is urgently required.

Studies have indicated that extracellular amyloid β (Aβ) plaques and intracellular tau neurofibrillary tangles (NFTs) are two neuropathological hallmarks of AD. Evidence suggests a crosslink between these two markers, although the detailed mechanisms remain unclear [5]. Aβ formation in amyloid precursor protein (APP) transgenic mice causes hyperphosphorylation of tau, leading to possible synaptic dysfunction, and ultimately, neuronal loss [5]. Tau is a microtubule-associated protein that contributes to microtubule stabilization, neuronal cell growth, axon morphology, and transportation under physiological conditions [5]. However, in pathological disease, hyperphosphorylation of tau diminishes its ability to bind and stabilize microtubules, causing microtubule sabotage [4,6]. Several kinases and phosphatases majorly contribute to tau phosphorylation, such as glycogen synthase kinase 3β (GSK3β), cyclin-dependent kinase 5 (CDK5), mitogen-activated protein kinase (MAPK), and protein phosphatase 2A (PP2A), and are deemed potential therapeutic targets for AD [6].

Evodiamine, an indole alkaloid derived from the fruit of *Evodia rutaecarpa*, possesses several biological effects, such as anti-obese [7], anti-inflammatory [8], analgesic [9], antibacterial [10], antidepressive [11], and neuroprotective effects [12]. Furthermore, evodiamine can ameliorate the blood–brain barrier (BBB) permeability and improve cognitive impairment by attenuating cellular apoptosis [12,13], decreasing oxidative stress, and reducing inflammation [14] in ischemia and AD mouse models. Evodiamine can increase p-GSK3β Ser9 expression [12], an inhibited form of GSK3β, which is the major kinase causing tau phosphorylation [6]. However, whether evodiamine inhibits hyperphosphorylated tau and its self-aggregates to form NFTs resulting in neuronal cell apoptosis remains unclear. Therefore, we determined whether evodiamine decreases tau phosphorylation for a neuroprotective effect against AD.

## 2. Results

### 2.1. Evodiamine Significantly Inhibited Hyperphosphorylation of Tau

**Figure 1:**
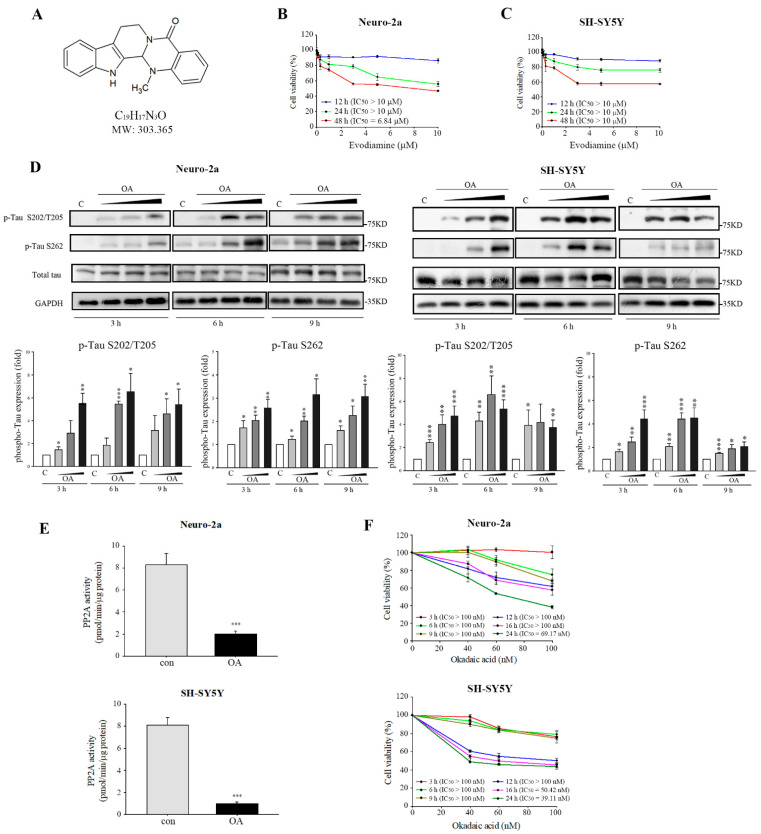
Okadaic acid (OA) induced tau phosphorylation. (**A**) Chemical structure of evodiamine. Neuro-2a (**B**) and SH-SY5Y cells (**C**) were incubated for 24 h with or without the indicated concentrations of evodiamine, and cell viability was determined by MTT assay. (**D**) Neuro-2a and SH-SY5Y cells were treated without or with OA (40, 60, 100 nM) for 3, 6, or 9 h. Cell lysates were subjected to Western blot analysis using the indicated antibodies. (**E**) Total extracts from cells treated with OA at 60 nM for 6 h; the PP2A activity was determined using a Ser/Thr protein phosphatase assay kit. (**F**) Cells were incubated for 3, 6, 9, 12, 16, or 24 h with or without the indicated concentrations of OA, and cell viability was determined by MTT assay. IC_50_ values were calculated by a sigmoidal dose-response equation. The results are shown as the mean ± SEM from three independent experiments (ANOVA, * *p* < 0.05, ** *p* < 0.01, and *** *p* < 0.001 vs. control).

Figure 1A depicts the structure of evodiamine. We first evaluated the cell viability inhibition of evodiamine in Neuro-2a and SY-SY5Y neuronal cells. The IC_50_ values of 12- or 24-h evodiamine treatment were >10 μM (Figure 1B,C); therefore, further studies were conducted under 10 μM. Significant tau phosphorylation was observed in Neuro-2a and SH-SY5Y cells by using a well-established tau phosphorylation-inducing agent, okadaic acid (OA), a polyether C_38_ fatty acid extracted from the black sponge *Hallichondria okadai*, to identify the inhibition of tau phosphorylation by evodiamine [15]. OA is a potent inhibitor of PP2A and can induce AD-like tau hyperphosphorylation in vitro and in vivo [12,16]. In this study, OA increased the expression of tau protein phosphorylated at Ser202/Thr205 and Ser262 in both cell types (Figure 1D) in a concentration-dependent manner; however, it did not increase the total tau levels. The expression of tau phosphorylation on Ser202/Thr205 and Ser262 at 6 h and 60 nM significantly increased (Figure 1D). Moreover, the PP2A activity markedly reduced (Figure 1E), with no cell toxicity observed in either cell types (Figure 1F). Therefore, we utilized OA treatment at 6 h and 60 nM for further study.

**Figure 2:**
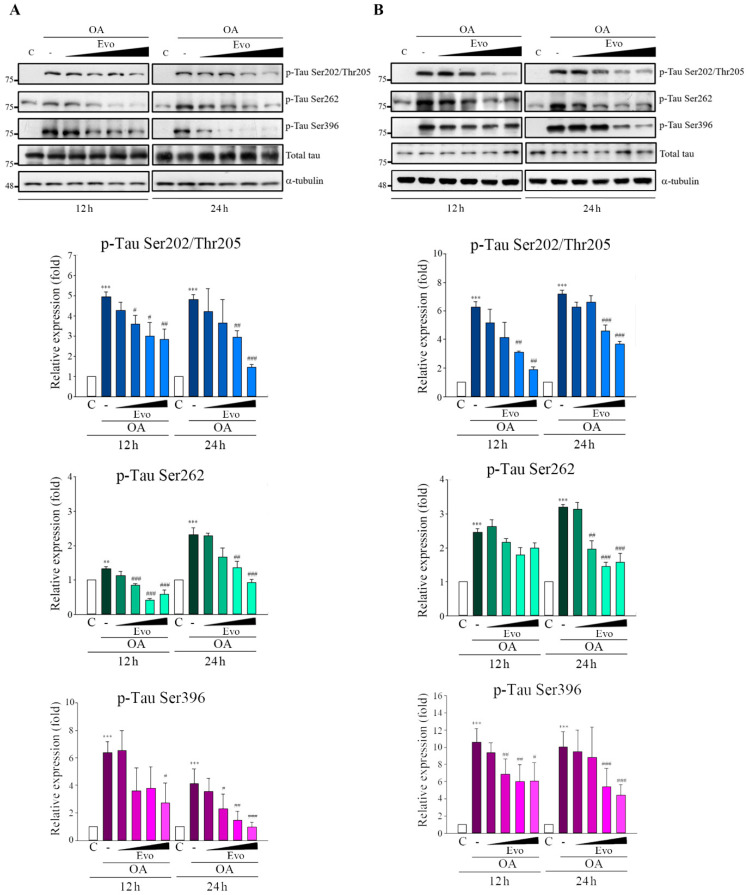
Evodiamine significantly inhibited OA-induced tau phosphorylation. Neuro-2a (**A**) and SH-SY5Y (**B**) cells were treated with evodiamine (0.1, 0.5, 1, 5 μM) for 12 or 24 h, then incubated with OA (60 nM) for another 6 h. Cell lysates were subjected to Western blot analysis using the indicated antibodies. Results are shown as the mean ± SEM from three independent experiments (ANOVA, ** *p* < 0.01, and *** *p* < 0.001 vs. control; # *p* < 0.05, ## *p* < 0.01, and ### *p* < 0.001 vs. relevant control).

Evodiamine significantly inhibited the phosphorylation of tau at Ser202/Thr205, Ser262, and Ser396 in Neuro-2a (Figure 2A) and SH-SY5Y (Figure 2B) cells in response to OA treatment in a concentration-dependent manner at 12 and 24 h, but total tau levels were unchanged. Consequently, evodiamine treatment inhibited OA-induced hyperphosphorylation of tau.

### 2.2. Studies on Mechanism Underlying Phosphorylated Tau Inhibition in Response to Evodiamine

**Figure 3:**
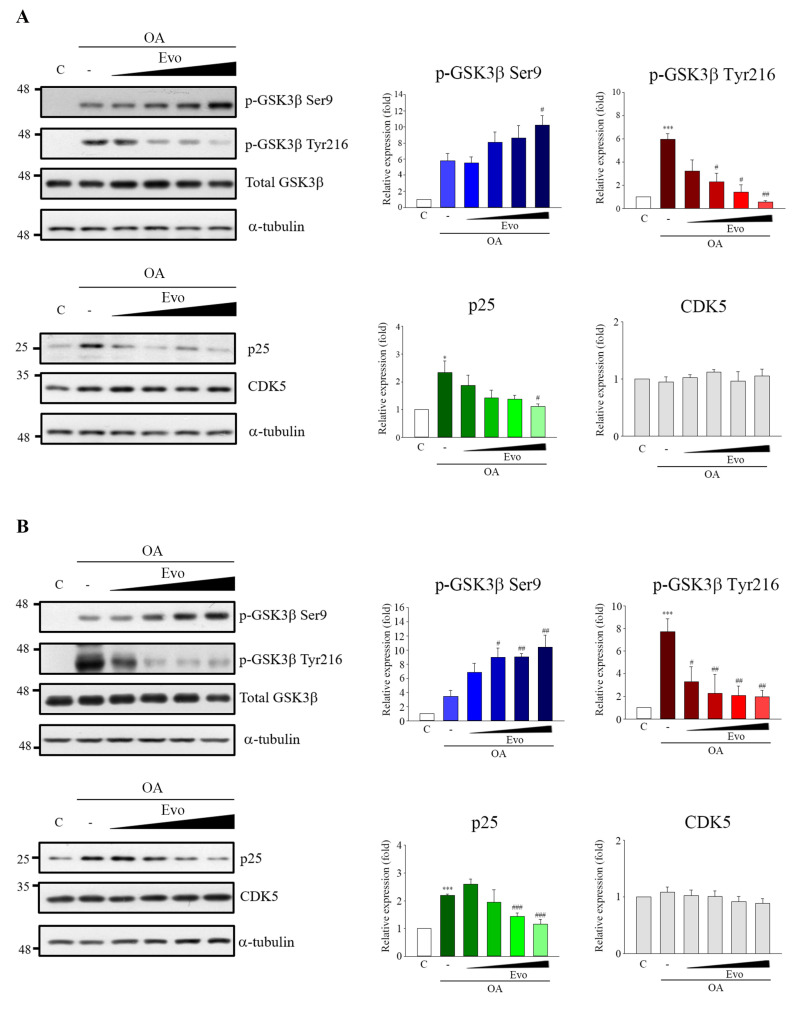
Evodiamine inhibited OA-induced hyperactivation of GSK3β and CDK5. Neuro-2a (**A**) and SH-SY5Y (**B**) cells were incubated with evodiamine (0.1, 0.5, 1, 5 μM) for 12 h, and then with OA (60 nM) for a further 6 h. Cell lysates were prepared for Western blot analysis of the indicated proteins. Results are shown as mean ± SEM from three independent experiments (ANOVA, * *p* < 0.05 and *** *p* < 0.001 vs. control; # *p* < 0.05, ## *p* < 0.01, and ### *p* < 0.001 vs. relevant control).

GSK3β and CDK5 are two major kinases in aberrant tau hyperphosphorylation [6]. GSK3β and CDK5 activity are increased in the brains of patients with AD [17,18], whereas over-activation of both in mice leads to tau hyperphosphorylation and an AD-like tau pathology [18]. GSK3β activity is increased by Tyr216 (active form) phosphorylation or decreased by Ser9 (inactive form) phosphorylation [19], and CDK5 activity is peculiarly increased by p25 linking [20]. Thus, we identified whether inhibition of tau phosphorylation by evodiamine occurs through activity modulation of GSK3β and CDK5. OA treatment in Neuro-2a (Figure 3A) and SH-SY5Y (Figure 3B) cells significantly increased GSK3β phosphorylation on Tyr216 and p25 expression; however, no significant changes in CDK5 levels were observed. Evodiamine treatment not only significantly decreased GSK3β phosphorylation on Tyr216 but also increased GSK3β phosphorylation on Ser9 as well as decreased the p25 expression. However, no obvious changes in GSK3β and CDK5 levels were observed in both cell types (Figure 3A,B).

**Figure 4:**
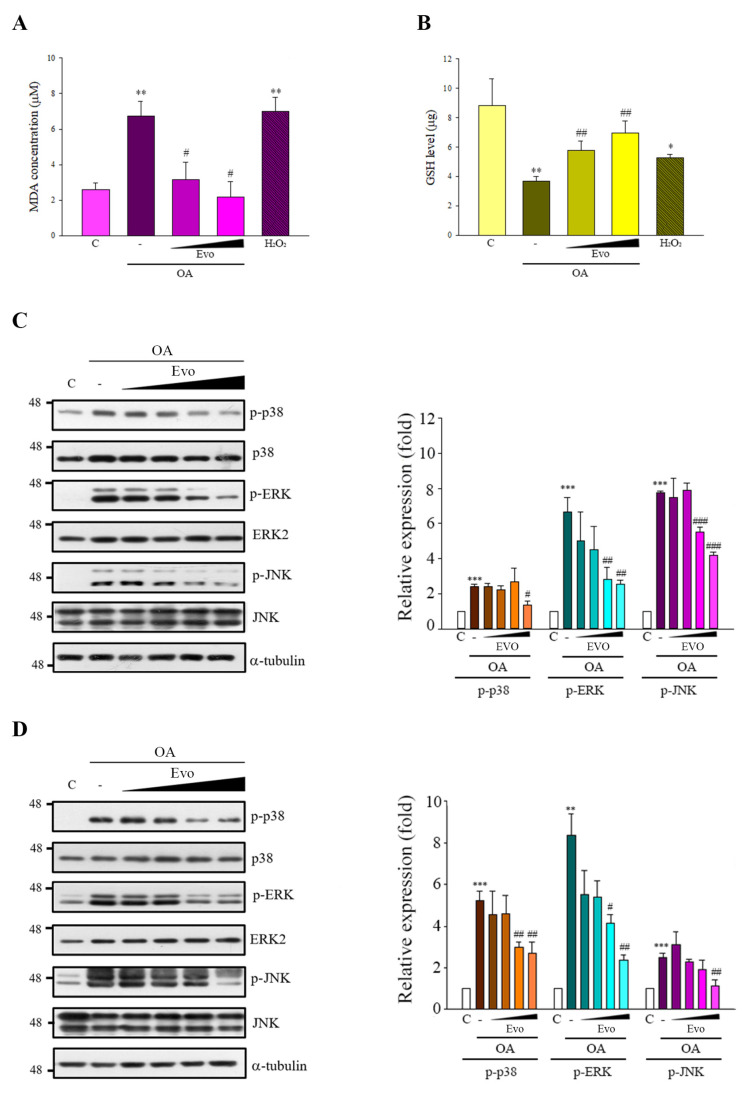
Evodiamine significantly inhibited OA-induced oxidative stress and MAPK activity. (**A**,**B**) SH-SY5Y cells were treated with evodiamine (0.5 or 1 μM) for 12 h, and then with OA (100 nM) for a further 5 h, or H_2_O_2_ (150 μM) for 5 h as a positive control. Malondialdehyde (MDA) (**A**) and glutathione (GSH) (**B**) levels were detected. (**C**,**D**) Neuro-2a (**C**) and SH-SY5Y (**D**) cells were incubated with evodiamine (0.1, 0.5, 1, 5 μM) for 12 h, and then with OA (60 nM) for a further 6 h, after which the cell lysates were subjected to immunoblotting. Results are shown as the mean ± SEM from three independent experiments (ANOVA, * *p* < 0.05, ** *p* < 0.01, and *** *p* < 0.001 vs. control; # *p* < 0.05, ## *p* < 0.01, and ### *p* < 0.001 vs. relevant control).

Studies have demonstrated that OA also generates intracellular reactive oxygen species (ROS) and then activates MAPKs signals, contributing to tau phosphorylation and cellular apoptosis [21]. Here, OA treatment increased oxidative stress, characterized by increased malondialdehyde (MDA) (Figure 4A) and decreased glutathione (GSH) levels (Figure 4B). OA treatment also triggered phosphor-p38, phosphor-ERK, and phosphor-JNK in both cells (Figure 4C,D)

### 2.3. Neuroprotective Effects of Evodiamine

**Figure 5:**
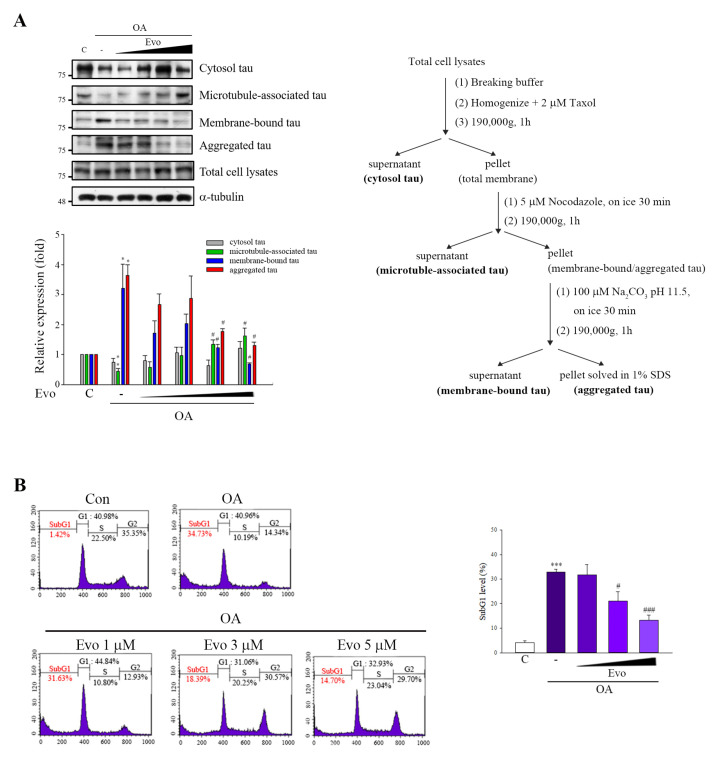
Evodiamine significantly attenuated tau aggregation and neuronal cell death. (**A**) SH-SY5Y cells were pretreated with evodiamine (0.1, 0.5, 1, 5 μM) for 12 h, and then incubated with OA (60 nM) for a further 6 h. The fractionation scheme used to separate different cellular pools of tau and the Western blot analysis of the effects of the compounds on tau pools generated using the fractionation scheme are presented. (**B**) SH-SY5Y cells were treated with evodiamine (1, 3, 5 μM) for 12 h and then treated with OA (100 nM) for another 12 h. The cells were fixed and stained with propidium iodide and analyzed by flow cytometry. Percentages of the sub-G1 phase in response to drug treatment; results are shown as the mean ± SEM from three independent experiments (ANOVA, * *p* < 0.05, ** *p* < 0.01, *** *p* < 0.001 vs. control; # *p* < 0.05 and ### *p* < 0.001 vs. relevant control).

The formation of NFTs with hyperphosphorylated tau is a primary pathological component of AD [3]; thus, removal of tau is considered a relevant therapeutic strategy. We used an established method to evaluate whether evodiamine suppresses the polymerization of phosphorylated tau [22]. The levels of tau in the membranes and aggregates significantly increased and those of microtubule-associated tau decreased in OA-treated SH-SY5Y cells. Evodiamine treatment downregulated aggregated tau and membrane-bound tau levels, recovering the microtubule-associated tau levels (Figure 5A). We examined whether evodiamine prevents neuronal cells from apoptosis; flow cytometry revealed that evodiamine decreased the sub-G1 population (Figure 5B), suggesting its neuroprotective effects.

**Figure 6:**
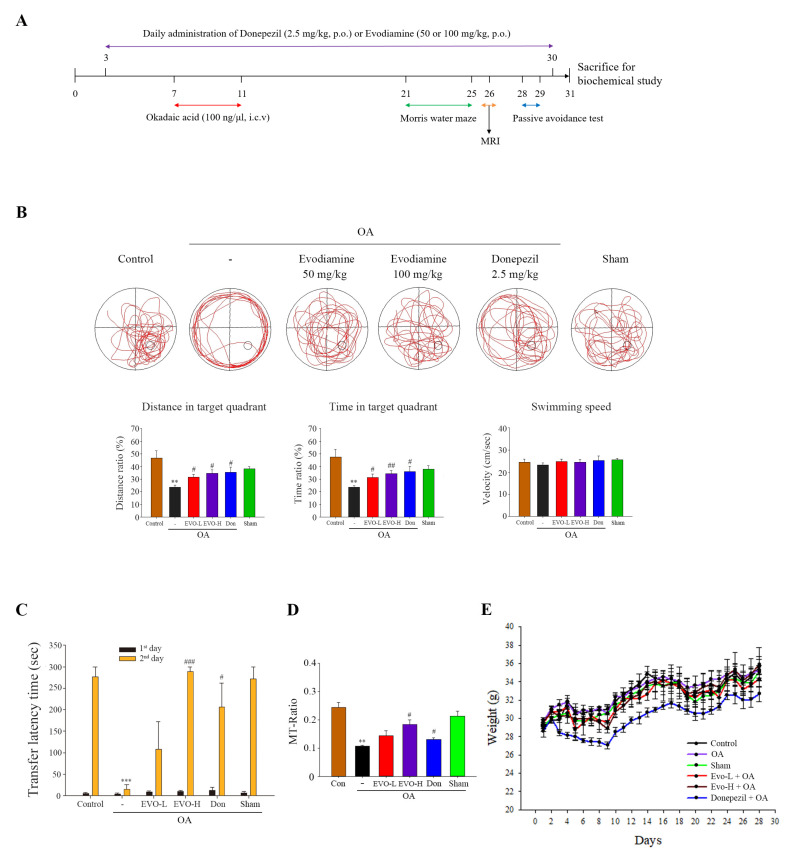
Evodiamine significantly ameliorated spatial memory impairment in vivo. (**A**) Experimental design. (**B**) Representative behavioral traces, swimming distance, time spent in target quadrant, and swimming speed were estimated using a Morris water maze. (**C**) Representative behavior traces and time taken by each animal to move from the light compartment to the dark compartment within 300 s in a passive avoidance test. (**D**) Magnetization transfer-ratios (MT-Ratios) in the hippocampus of the mice were measured by magnetic resonance imaging (MRI). (**E**) The weights of the animals were measured during test periods. Results represent the mean ± SEM (*n* = 5) (ANOVA, ** *p* < 0.01 and *** *p* < 0.001 vs. control; # *p* < 0.05, ## *p* < 0.01, and ### *p* < 0.001 vs. OA-treated).

We investigated whether evodiamine improves impaired spatial memory and learning using the Morris water maze test in mice receiving OA-intracerebroventricular (ICV) injection. Donepezil improves cognition in AD patients and was used as a positive control. OA-treated mice demonstrated an irregular travel path to find the platform and spent less distance, movement, and time in the target quadrant than control and sham groups (Figure 6B). Furthermore, there was no significant difference in the distance, movement, and time spent in the target quadrant between the control and sham group, indicating that ICV injection did not cause spatial memory impairment. Evodiamine-treated mice spent a longer distance, movement, and time on the target quadrant; the movement velocity was unchanged for each group (Figure 6B). We also examined emotional learning and memory using the passive avoidance test. The transfer latency of the OA-treated group was significantly shorter than that of the control and sham groups in the day 2 trial; transfer latency time did not differ between the control and sham groups (Figure 6C). Evodiamine treatment significantly increased the transfer latency time compared with the OA group, and evodiamine (100 mg/kg, high dose)-treated mice manifested a longer transfer latency time than the donepezil-treated group (Figure 6C). Studies have also demonstrated reduction in the magnetization transfer ratio (MTR) in the brains of patients with AD, suggesting less remaining brain tissue in AD patients; thus, the MTR might be a sensitive tool for assessing tissue damage in AD [23]. OA-treated mice exhibited a significant reduction in MTR, whereas evodiamine (100 mg/kg, high dose)-treated mice had a significantly increased MTR, indicating that evodiamine recovered the OA-induced decline in the hippocampus (Figure 6D). Further, the administration of evodiamine caused no significant body weight changes compared with the control group (Figure 6E).

**Figure 7:**
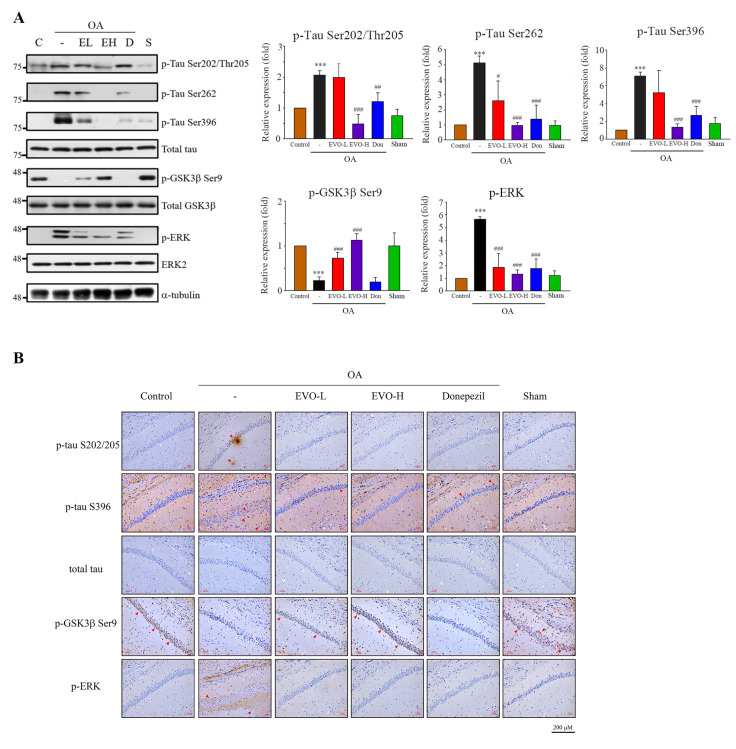
Evodiamine significantly downregulated the phosphorylation of tau, GSK3β, and ERK in the mouse brain. Institute Cancer Research ICR mice (6 weeks of age) were treated (Figure 6); the mice were sacrificed, and the brain was removed for Western blot analysis of tau phosphorylation on Ser202/Thr205, Ser262, Ser396, GSK3β phosphorylation of Ser 9, and ERK phosphorylation (**A**) and immunohistochemical analysis in the hippocampal CA1 region (magnification ×200, insert ×400) (**B**). Red arrowheads show the phosphorylated tau, GSK3β, and ERK proteins. Scale bar = 200 μm. Results represent the mean ± SEM (ANOVA, *** *p* < 0.001 vs. control; # *p* < 0.05, ## *p* < 0.01, and ### *p* < 0.001 vs. OA-treated).

We next identified whether evodiamine downregulates tau phosphorylation in the brain. The results of Western blotting (whole brain) and immunohistochemical analysis of the CA1 region indicated that OA treatment not only triggered significant phosphorylation of tau on Ser202/Thr205, Ser262, Ser396, and phosphor-ERK but also downregulated p-GSK3β on Ser9 (Figure 7A,B). Evodiamine treatment inhibited tau phosphorylation on multiple sites, ERK phosphorylation, and increased the phosphorylation of GSK3β on Ser9 in the mouse brain.

**Figure 8:**
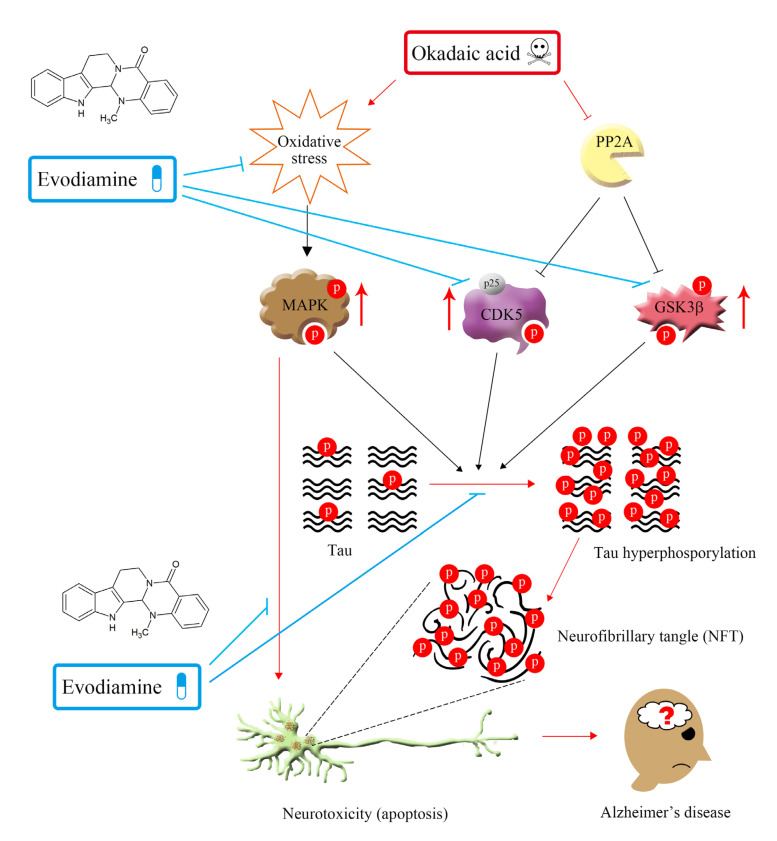
Summary of the proposed mechanism of the neuroprotective effect of evodiamine. P: phosphorylation.

A summary of the proposed neuroprotective mechanism of evodiamine is illustrated in Figure 8.

## 3. Discussion

Studies indicated that evodiamine can inhibit streptozotocin-triggered oxidative stress and inflammatory cytokines (e.g., TNF-α, IL-1β, and IL-6) in the hippocampi of mice [14], and it shows high permeability through the BBB for neuroprotective effects by reducing 1-methyl-4-phenylpyridinium ion (MPP^+^) or hydrogen peroxide-induced injury [16]. Furthermore, 20 μM treatment of evodiamine had no significant effect on cell viability in mouse microglial BV-2 cells and mouse hippocampal neuronal HT22 cells [13,24]. These findings suggest that evodiamine is therapeutic for neurodegenerative diseases. Zhang’s group demonstrated that evodiamine significantly increased the serum levels of acetylcholine and decreased the levels of acetylcholinesterase in the serum, hypothalamus, and brain, by reducing the Aβ_42_ deposition in the brain [13]. Wang et al. also indicated that evodiamine inhibits glial cell activation and neuroinflammation in the hippocampus [14]. Several protein kinases, including GSK3β, CDK5, and MAPKs, can phosphorylate tau proteins at various sites, triggering tau hyperphosphorylation, whereas its dephosphorylation is catalyzed by protein phosphatases, of which, PP2A accounts for 70% of human brain tau phosphatase activity [6]; the imbalance between tau phosphorylation and dephosphorylation is critical to AD tauopathy. OA treatment not only inhibited PP2A activity but also induced an Alzheimer-like hyperphosphorylation and accumulation of tau through increased activity of GSK3β and CDK5 both in vivo and in vitro [25]. Here, OA-treated neuronal cells significantly increased tau phosphorylation (Figure 3) and hyperphosphorylated tau aggregation, leading to neuronal death (Figure 5), consistent with the findings of previous studies. Evodiamine treatment significantly decreased phosphorylated (Figure 3) and aggregated tau and downregulated apoptosis (Figure 5). Evodiamine treatment ameliorated learning and memory impairments in vivo (Figure 6), emphasizing its therapeutic potential for AD.

GSK3β and CDK5 are major kinases in aberrant tau phosphorylation. GSK3β is a constitutively active protein kinase and its kinase is primarily regulated via the phosphorylation inhibition on Ser9 and phosphorylation activation on Tyr216 [19]. GSK3β-mediated phosphorylation of tau primarily occurs in the regions surrounding the microtubule-binding domain, whereas phosphorylation at these sites has been found to cause tau detachment from microtubules, resulting in self-aggregation [26]. Increased GSK3β activity has been found in AD patients [17], and overexpression of GSK3β in mice results in AD-like tau pathology [27]. CDK5 activity is closely linked with p35/25 protein expression, and CDK5, along with its major activator p35, is involved in neuronal cellular functions; however, under neurotoxic stress, the activating calpain which cleaves p35 to produce p25. CDK5 combined with p25, causing the hyperactivation of CDK5, leads to tau phosphorylation [28]. Here, evodiamine treatment not only significantly decreased GSK3β phosphorylation on Tyr216, but also increased GSK3β phosphorylation on Ser9 and decreased the expression of p25 in an OA-induced model (Figure 3). Thus, evodiamine inhibits tau phosphorylation by modulating GSK3β and CDK5 kinase activity. Furthermore, ROS regulates signaling in cell proliferation and apoptosis, whereas activation of MAPKs contributes to ROS-induced signal pathways [29]. OA treatment increases ROS levels and induces MAPK activation, causing tau phosphorylation at sites in paired helical filament (PFH)-tau, ultimately causing apoptosis [21]. In this study, evodiamine treatment significantly reversed OA treatment-induced GSH reduction and MDA increase, along with significantly increased MAPKs activation (Figure 4). Collectively, our results indicated that evodiamine decreased tau phosphorylation, exerting neuroprotective effects against AD via the inhibition of GSK3β, CDK5, and MAPKs signals.

## 4. Materials and Methods

### 4.1. Materials

Evodiamine was obtained from Matsuura Yakugyo Co Ltd (Nagoya, Japan). Primary antibodies against APP, p-GSK3β (Ser9), p-GSK3β (Tye216), p-SAPK/JNK (Thr183/Tyr185), p-p44/42 MAPK (ERK1/2) (Thr202/Tyr204), and p-p38 MAPK (Thr180/Tyr182) were purchased from Cell Signaling Technology (Danvers, MA, USA). Antibodies against p-tau (Ser396), JNK, ERK, and p38 were purchased from Abcam (Cambridge, MA, USA). Antibodies against p-tau (Ser202/Thr205), p-tau (Ser262), and tau were obtained from Thermo Fisher Scientific (Waltham, MA, USA); antibodies to GSK3β and GAPDH were obtained from GeneTex Inc. (Hsinchu city, Taiwan). Horseradish peroxidase (HRP)-conjugated antimouse and antirabbit IgG secondary antibodies were purchased from Jackson ImmunoResearch Inc. (West Grove, PA, USA). OA was purchased from Cayman Chemical Company (Ann Arbor, MI, USA). Unless otherwise stated, all other chemicals were purchased from Sigma-Aldrich (St. Louis, MO, USA).

### 4.2. Cell Culture

Human neuroblastoma cell line SH-SY5Y was kindly provided by Prof. Shiow-Lin Pan (Taipei Medical University) and was maintained in Ham’s F12 nutrient mixture/minimum essential media with 10% fetal bovine serum, penicillin (100 units/mL), and streptomycin (100 μg/mL). The mouse neuroblastoma cell line Neuro-2a was purchased from the Bioresource Collection and Research Center (Hsinchu, Taiwan) and cultured in minimum essential media containing 10% fetal bovine serum, penicillin (100 units/mL), and streptomycin (100 μg/mL). All cell lines were incubated at 37 °C with 5% CO_2_.

### 4.3. PP2A Activity

PP2A activity was assessed using a serine/threonine phosphatase assay system (Promega Corp., Madison, WI, USA). An end product of lipid peroxidation, MDA, was spectrophotometrically measured according to the assay protocol (Cell Biolabs, San Diego, CA, USA). Endogenous antioxidant glutathione levels were measured using a glutathione colorimetric assay kit (BioVision Inc., Milpitas, CA, USA).

### 4.4. Estimation of Lipid Peroxidation

An end product of lipid peroxidation, MDA, was measured spectrophotometrically at a wavelength of 532 nm using malondialdehyde bis(dimethyl acetal) as a standard as per the assay protocol (Cell Biolabs, San Diego, CA, USA).

### 4.5. Estimation of Glutathione

Endogenous antioxidant glutathione levels were estimated using a glutathione colorimetric assay kit (BioVision Inc., Milpitas, CA, USA) by reacting it with 5,5′-dithiobis 2-nitrobenzoicacid, using reduced glutathione as a standard at a 412 nm wavelength.

### 4.6. Flow Cytometry

After treatment, the cells were collected, washed with cold phosphate-buffered saline (PBS), and fixed with 75% alcohol overnight at −20 °C. After centrifugation, fixed cells were washed with cold PBS and resuspended in DNA extraction buffer (0.2 M Na_2_HPO_4_, 0.1 M citric acid, pH 7.8) for 30 min. Cells were then centrifuged and incubated with propidium iodide (PI) (0.1% Triton X-100, 100 μg/mL RNase A, and 80 μg/mL PI in PBS) for 30 min. A FACScan Flow cytometer and Cell Quest software (Becton Dickinson, Mountain View, CA, USA) was used to analyze the cell cycle.

### 4.7. Subcellular Fractionation

This assay followed a previously published method [22]. Briefly, cells (1 × 10^7^) were treated with drugs and scraped off into a lysis buffer (0.25 M sucrose, 10 mM HEPES, pH 7.2, 1 mM MgAc_2_, and protease inhibitors). Lysates were centrifuged at 190,000× *g* for 1 h, and the supernatant was collected as the cytosolic fraction. The pellet was resuspended and incubated with 5 μM nocodazole on ice for 30 min, and then centrifuged for 1 h at 190,000× *g*. The supernatant and pellets contained microtubule-tau and membrane-bound and aggregated tau, respectively. The pellets were extracted using 100 mM sodium carbonate buffer, pH 11.5, centrifuged at 190,000× *g* for 1 h, and washed with 1% SDS to produce a fraction containing tau aggregates. Samples containing equal protein were analyzed using SDS-PAGE.

### 4.8. Immunoblot Analyses

For 10 min at 4 °C, 1 × 10^6^ cells were incubated in lysis buffer (20 mM HEPES, pH 7.4; 2 mM EGTA, 0.1% Triton X-100; 50 mM β-glycerophosphate; 1 mM DTT; 10% glycerol; 1 μg/mL leupeptin; 1 mM sodium orthovanadate; 1 mM phenylmethylsulfonyl fluoride, and 5 μg/mL aprotinin). Next, the cells were removed, placed on ice for 10 min, and subjected to 30 min centrifugation (17,000× *g*) at 4 °C. Next, we electrophoresed 20-μg protein samples on SDS polyacrylamide gels before transferring them onto a nitrocellulose membrane. The nitrocellulose membrane was subsequently blocked through 30 min incubation with 5% bovine serum albumin in Tris-buffered saline containing 0.1% Tween 20 (TBST) at room temperature (RT). Immunoblots were obtained through incubation overnight at 4 °C with primary antibodies in TBST and subsequent 1 h incubation at RT with secondary antibodies conjugated with HRP. Measurement of antibody binding was performed through photographic film exposure and application of an enhanced chemiluminescence reagent (GE Healthcare Corp., Buckinghamshire, UK).

### 4.9. Surgery and Microinjection for Intracerebroventricular (ICV) Administration of Okadaic Acid

The mice were anesthetized with Zoletil (I.M.) and restrained in a stereotactic apparatus (Stoelting Company, Wood Dale, Illinois, USA). A midline sagittal incision was made in the scalp. Okadaic acid (100 ng/1 μL in ACSF (147 mM NaCl, 2.9 mM KCl, 1.6 mM MgCl_2_, 1.7 mM CaCl_2_, and 2.2 mM dextrose) or only ACSF (1 μL) was ICV administered once into left lateral cerebral ventricle by microsyringe (Model 1701N, needle size 26s gauge) (Hamilton Company, NV, USA), using the coordinates: 0 mm posterior; −2.0 mm lateral; −2.5 mm ventral to bregma. After microsyringe puncture into cerebral ventricle and waiting for 5 min to obtain the equilibrium of brain pressure, microinjection continues for a period of 1 min (1 μL/min) by syringe pump (KD Scientific Inc., Holliston, MA, USA) then followed by an additional 5 min waiting time to allow for diffusion away from the injection site. Finally, the wound would be sutured rapidly.

### 4.10. Analysis of Cognitive Dysfunction

Six-week-old male ICR mice were randomly assigned to six groups (*n* = 5). The groups and treatment conditions are detailed in Table 1. Total experimental period was 31 d. Mice were intragastrically administered evodiamine (50, 100 mg/kg) or donepezil (2.5 mg/kg) with vehicle (0.9% saline, including 1% DMSO and 0.5% tween-80) once daily from day 3–30. On days 7–11, mice were treated with OA (100 ng) dissolved in sterile artificial cerebrospinal fluid by intracerebroventricular (ICV) injection once into the left lateral cerebral ventricle. Ten days after ICV injection, mice were subjected to the Morris water maze (days 21–25), magnetic resonance imaging (MRI) analysis (day 26), and the passive avoidance test (days 28–29). On day 31, the mice were sacrificed, and their brains extracted to evaluate tau phosphorylation and immunohistochemistry stains.

### 4.11. Morris Water Maze

The water maze was a white circular pool filled with water that was maintained at 25 °C. A black platform was submerged 2 cm below the surface of the water, and Styrofoam beads were added to make the platform invisible. Animals were trained to remain on the platform for 10 s upon reaching it. If the mice failed to locate the platform in 120 s, they were placed on the platform for 10 s such that they could learn and memorize the location; training was performed twice daily over 4 days for 1 week.

### 4.12. Magnetic Resonance Imaging (MRI)

Mice were subjected to ^1^H MRI scanning using a 7T animal scanner (Biospec 70/30 AS, Bruker Biospin MRI, Ettlingen, Germany) with an actively shielded gradient (BGA-12S, maximum strength of 670 mT/m). A 20 mm ^1^H selective double-tuned surface coil was used for signal excitation and reception. A system that was interfaced to a Linux PC running Topspin 2.0 and Paravision 5.1 software was used to acquire data. Before MRI, the mice were anesthetized by administering a mixture of 97% oxygen and 3% isoflurane. The anesthesia concentration was maintained at a proportion of 98% oxygen and 2% isoflurane during MR acquisition. To ensure consistent slice positioning, the coronal multislice rapid acquisition and relaxation enhancement (RARE) images were first acquired in axial orientations with the following parameters: repetition time (TR) = 3000 ms, echo time (TE) = 33 ms, acquisition matrix size = 256 × 256, field of view (FOV) = 20 × 20 mm^2^, 9 slices, slice thickness = 1 mm, and RARE factor = 8. The MT images, −1 mm posterior to the bregma, were acquired using a multiecho sequence in axial orientation with the following parameters: TR = 3000 ms, TE = 33 ms, acquisition matrix size = 256 × 256, FOV = 20 × 20 mm^2^, slice thickness = 1 mm, and number of averages = 1. RARE images were acquired without an off-resonance radiofrequency (RF) pulse (unsaturated) and with an off-resonance RF pulse (saturated) at an offset frequency of −16,875 Hz (pulse strength = 12 μT, number of pulses = 36, pulse length = 40 ms, saturation time = 1440 ms). The MT-ratio was calculated using the voxel extent ratio of the signal intensities by Image J. MRI analysis was entrusted by the Instrumentation Center of National Taiwan University.

### 4.13. Passive Avoidance Test

The passive avoidance test used two equally sized compartments (17 × 12 × 10 cm) set up with an electrifiable grid floor and separated by a guillotine door. On day 1, mice were initially placed in the light compartment to acclimatize for 40 s. The door was automatically opened and closed after the mice entered the dark compartment and were given a low-intensity electric foot shock (0.5 mA, 3 s) in the dark compartment. The transference of mice from the light to dark compartments was recorded in seconds as the transfer latency time. On day 2, no foot shock was delivered, and the duration of the trial was 300 s to examine the transfer latency time. The transfer latency time was measured using the Gemini Avoidance System (San Diego Instruments, San Diego, CA, USA).

### 4.14. Immunohistochemical Staining

On day 31, the mice were anesthetized and sacrificed by means of perfusion with 40–50 mL PBS, followed by 40–50 mL 4% paraformaldehyde in PBS as a fixative solution. Subsequently, the brains were removed and soaked in the same fixative solution. The immunohistochemistry stains were entrusted by Rapid Science Co. Ltd. (Taichung, Taiwan). Briefly, the whole brains were dehydrated, followed by immersion in paraffin and subsequently cut into 5 μm thick coronal slices. Replacing paraffin with xylene for 10 min dewaxes paraffin slices, which were then treated by 95%, 85%, 75% alcohol and subsequently distilled water for 5 min. The slices were immersed in Trilogy solution at 121 °C for 10 min to conduct antigen presentation, and cooled down naturally at RT. After washing three times with PBS for 3 min, the slices were placed in 3% H_2_O_2_ solution for 10 min at RT to reduce endogenous peroxidase activity, then washed three times with PBS for 3 min. The slices were incubated with 5% fetal bovine serum for 1 h at RT and then with primary antibody in 5% FBS overnight at 4 °C. After incubation, the slices were rinsed with PBS and reacted with HRP polymer conjugate reagent for 1 h at RT, then the slices were treated by DAB chromogen reagent to develop color.

### 4.15. Data Analysis and Statistics

Data are expressed as mean ± SEM and were analyzed using one-way ANOVA. When ANOVA showed significant differences between groups, the Tukey’s post hoc test was used to determine the pairs of groups showing statistically significant differences. Parameters with *p* < 0.05 were considered statistically significant.

## 5. Conclusions

Evodiamine treatment significantly inhibited tau phosphorylation on Ser202/Thr205, Ser262, and Ser396 and downregulated tau aggregation and neuronal cell apoptosis. Evodiamine treatment ameliorated learning and memory impairments in vivo, downregulated tau phosphorylation, and attenuated neuronal loss in mice brains. Our novel findings indicate that evodiamine possesses neuroprotective effects against okadaic-acid-induced tau pathology.

## Figures and Tables

**Table 1:** Groups and treatment conditions.

Group Treatment	Oral (d 3–30) ^a^	ICV Injection (d 7–11) ^b^
Control	Vehicle ^c^	No treatment
Sham	Vehicle ^c^	Sterile artificial cerebrospinal fluid (ACSF) 1 μL
Okadaic acid	Vehicle ^c^	Okadaic acid 100 ng/1 μL ACSF
50 mg/kg Evodiamine	50 mg/kg evodiamine (vehicle) ^c^	Okadaic acid 100 ng/1 μL ACSF
100 mg/kg Evodiamine	100 mg/kg evodiamine (vehicle) ^c^	Okadaic acid 100 ng/1 μL ACSF
Donepezil	2.5 mg/kg donepezil (saline)	Okadaic acid 100 ng/1 μL ACSF

^a^ Administered once daily from the 3rd to the 30th day; ^b^ administered ICV injection only once on d 7–11; ^c^ vehicle components were described in experimental section.
